# Integration of Stable Ionic Liquid-Based Nanofluids into Polymer Membranes. Part II: Gas Separation Properties toward Fluorinated Greenhouse Gases

**DOI:** 10.3390/nano11030582

**Published:** 2021-02-26

**Authors:** Fernando Pardo, Sergio V. Gutiérrez-Hernández, Carolina Hermida-Merino, João M. M. Araújo, Manuel M. Piñeiro, Ana B. Pereiro, Gabriel Zarca, Ane Urtiaga

**Affiliations:** 1 Department of Chemical and Biomolecular Engineering, Universidad de Cantabria, 39005 Santander, Spain; pardof@unican.es (F.P.); gutierrezsv@unican.es (S.V.G.-H.); zarcag@unican.es (G.Z.); 2 Centro de Investigaciones Biomédicas (CINBIO), Departamento de Física Aplicada, Universidade de Vigo, 36310 Vigo, Spain; cahermida@uvigo.es (C.H.-M.); mmpineiro@uvigo.es (M.M.P.); 3 LAQV, REQUIMTE, Departamento de Química, Faculdade de Ciências e Tecnologia, Universidade Nova de Lisboa, 2829-516 Caparica, Portugal; jmmda@fct.unl.pt (J.M.M.A.); anab@fct.unl.pt (A.B.P.)

**Keywords:** ionanofluid, mixed-matrix membrane, poly(ether-block-amide), global warming, fluorinated refrigerant, R32 recovery, R410A

## Abstract

Membrane technology can play a very influential role in the separation of the constituents of HFC refrigerant gas mixtures, which usually exhibit azeotropic or near-azeotropic behavior, with the goal of promoting the reuse of value-added compounds in the manufacture of new low-global warming potential (GWP) refrigerant mixtures that abide by the current F-gases regulations. In this context, the selective recovery of difluorometane (R32, GWP = 677) from the commercial blend R410A (GWP = 1924), an equimass mixture of R32 and pentafluoroethane (R125, GWP = 3170), is sought. To that end, this work explores for the first time the separation performance of novel mixed-matrix membranes (MMMs) functionalized with ioNanofluids (IoNFs) consisting in a stable suspension of exfoliated graphene nanoplatelets (xGnP) into a fluorinated ionic liquid (FIL), 1-ethyl-3-methylpyridinium perfluorobutanesulfonate ([C_2_C_1_py][C_4_F_9_SO_3_]). The results show that the presence of IoNF in the MMMs significantly enhances gas permeation, yet at the expense of slightly decreasing the selectivity of the base polymer. The best results were obtained with the MMM containing 40 wt% IoNF, which led to an improved permeability of the gas of interest (*P_R32_ =* 496 barrer) with respect to that of the neat polymer (*P_R32_* = 279 barrer) with a mixed-gas separation factor of 3.0 at the highest feed R410A pressure tested. Overall, the newly fabricated IoNF-MMMs allowed the separation of the near-azeotropic R410A mixture to recover the low-GWP R32 gas, which is of great interest for the circular economy of the refrigeration sector.

## 1. Introduction

Over the years, the refrigeration industry has undergone very significant changes mainly directed towards the use of new refrigerant compounds with lower environmental impact. Under the Montreal Protocol, ratified in 1987, a plan was established to eliminate the use and marketing of chlorofluorocarbons (CFCs) and hydrofluorocarbons (HCFCs), the first and second generation of fluorinated gases (F-gases), because of their proven potential to destroy stratospheric ozone [1,2]. Since then, hydrofluorocarbons (HFCs), the third generation of F-gases, with no impact on stratospheric ozone have been massively used. However, HFCs still exhibit high global warming potential (GWP), which can be several orders of magnitude above that CO_2_ equivalent (GWP = 1). Currently, a new generation of F-gases has emerged owing to recent bans on the use of HFC refrigerants [3,4], namely, the hydrofluoroolefins (HFOs). Due to their zero-ozone depletion potential (ODP) and extremely low GWP—very similar to that of CO_2_—HFOs are starting to be used as environmentally friendly substitutes for HFC refrigerants either as pure compounds or in HFC/HFO mixtures with moderate GWP [5].

Despite these developments, the refrigeration industry still needs to adapt to more sustainable models aligned with the circular and low carbon economy. For this purpose, the novel F-gas regulations demand the implementation of new solutions that facilitate the recovery, recycling, and reuse of F-gases in order to reduce emissions and improve the management of refrigeration equipment at the end of its operational lifespan. In this context, technologies focusing on advanced gas separations are expected to assume a leading role in promoting and boosting the recovery of F-gases from mixtures that are on the verge of being phased out. One of the gases of greatest interest is the HFC R32 (difluoromethane, GWP = 677), which is found in an equimass proportion together with R125 (pentafluoroethane, GWP = 3170) in the widely employed mixture R410A (GWP = 1924). The interest in recovering R32 arises from its use in the formulation of new low-GWP HFC/HFO mixtures, such as R454B (GWP = 467) or R455A (GWP = 146) [6,7,8]. However, the separation of R410A into its main constituents entails serious challenges given that they form a near-azeotropic mixture with an azeotropic mixture at 91 mol% R32.

At present, there is a growing number of publications dealing with the separation of close-boiling point and azeotropic refrigerant gas mixtures. Most of them rely on the use of advanced materials, such as ionic liquids [9,10,11,12,13,14,15,16] or deep eutectic solvents [17], as selective absorbent species, and porous materials such as activated carbons [18], zeolites [19,20] or metal organic frameworks (MOFs) [21] as selective adsorbents. Moreover, regarding membrane technology, we have recently analyzed for the first time the potential of several poly(ether-b-amide) membranes, with trade name Pebax^®^, to separate HFC/HFO mixtures [22], and reported an improved separation performance of composite Pebax^®^1657 ionic liquid polymer membranes (CILPMs) to selectively recover R32 from the mixture R410A [23]. The implementation of membrane-based separations entails well-known advantages such as low energy requirements, no phase change, low capital costs and ease of operation and scalability [24,25,26], yet there is still a significant scarcity of knowledge on its application towards the recovery of value-added F-gases.

Rubbery Pebax^®^ copolymers present a series of functional features that make them viable candidates for the separation of high-sorbing penetrants such as CO_2_, light hydrocarbons or organic vapors [27,28,29,30,31]. Moreover, Pebax^®^1657 displays a high degree of compatibility with many additives that facilitate the synthesis of Pebax^®^-based hybrid membranes with improved separation performance, which indeed can be easily manufactured through conventional solvent casting techniques [32,33]. Apart from the previously mentioned CILPMs, one type of Pebax^®^-based hybrid membranes whose popularity has increased significantly over the last decade are the mixed-matrix membranes (MMMs), which consist of inorganic particles dispersed inside the polymer matrix at nanometric scale and whose aim is to combine the improved gas transport properties of inorganic particles with the easy and cost-effective processing of polymeric materials. In this regard, the nature of the inorganic fillers employed for the functionalization of Pebax^®^ MMMs is highly diverse; the most common being zeolites [34], MOFs [35,36], zeolitic imidazolate frameworks (ZIFs) [37], pristine graphene [38], graphene oxide [39], or metal and metal-oxide nanoparticles [40,41], among others.

Given the promising results obtained with MMMs, mainly focused on CO_2_ selective separation [42,43,44,45,46], in this work we explore for the first time the use of MMMs functionalized with exfoliated graphene nanoplatelets (xGnP) for the separation of HFCs. In this sense, the role of pristine graphene in this type of applications, given its bidimensional structure and the fact that it is impermeable to gases, is to generate some degree of tortuosity within the polymer matrix and thus, modify the path of the gas molecules during permeation [47,48]. Nonetheless, the integration of this type of inorganic filler in the Pebax^®^ matrix may present several drawbacks, such as inappropriate adhesion between the fillers and the polymer or rigidification of the polymer segments around the graphene particles. Different approaches can be followed to tackle these issues, among them, the addition of xGnP can be facilitated by preparing the so-called ioNanofluids (IoNF) [49]. IoNF are stable suspensions prepared by dispersing the xGnP into an appropriate ionic solvent. In the present study we selected the fluorinated ionic liquid (FIL) 1-ethyl-3-methylpyridinium perfluorobutanesulfonate [C_2_C_1_py][C_4_F_9_SO_3_]. Thanks to the perfluorinated nature of the anion, the selected FIL is expected to play multifold functions. First, in accordance with its good surfactant behavior [49,50,51], the [C_4_F_9_SO_3_] anion has the ability to facilitate the exfoliation and dispersion of xGnP in the polymeric matrix and acts as void filler around interstitial areas between the polymer molecules and the xGnP. In addition, given the high absorption and permeation performance of several greenhouse gases in this FIL [12,52], remarkable improvements are expected in terms of F-gas solubility and permeability through hybrid membranes based on this FIL.

Therefore, in the present study, the separation of the constituents of the R410A mixture, i.e., R32 and R125, is evaluated for the first time under continuous mixed gas permeation conditions through Pebax^®^1657 MMMs containing an IoNF constituted by pristine xGnP and the FIL [C_2_C_1_pyr][C_4_F_9_SO_3_]. The F-gas permeation properties are determined at several feed pressures. Moreover, to evaluate the effect of xGnP addition, different membranes are tested by modifying the polymer/IoNF mass proportions as well as the concentration of xGnP in the IoNF (1, 10 and 20 wt%). Finally, R32 and R125 solubility isotherms are determined for the best-performing membrane with and without xGnP and compared to the solubility of these gases in neat Pebas1657 to assess the influence of the xGnP content on the transport properties.

## 2. Materials and Methods

### 2.1. Reagents and Membrane Separation

Butan-1ol (99.9 wt%), purchased from VWR (Barcelona, Spain), was used as solvent for the preparation of all dense films. The block copolymer poly(ether-block-amide) Pebax1657^®^MH grade was kindly provided as pellets by Arkema Química S.A. (Barcelona, Spain). It consists of soft and flexible polyether blocks (PEO) interlinked with hard and rigid polyamide 6 (PA6) segments.

The FIL [C_2_C_1_py][C_4_F_9_SO_3_] (≥97 wt%), supplied by IoLiTec (Heilbronn, Germany), was used for the preparation of the CILPMs and the synthetized [C_2_C_1_py][C_4_F_9_SO_3_]-based IoNF was employed for the preparation of the MMMs. For membrane preparation, all reagents (solvent, polymer, FIL, and IoNF) were used as received without any further purification step. Accordingly, general properties of the polymer and FIL used are summarized in Table 1 and Table 2, respectively.

For membrane preparation, near 3 wt% of polymer pellets were dissolved in butan-1-ol at 100 °C under magnetic stirring. Once the polymer was dissolved, the adequate amount of either FIL or IoNF was added and the mixture was stirred at 100 °C for 1 h to ensure homogenization and to avoid gelation of the mixture. Afterwards, the solution was poured onto a glass Petri dish, and the solvent was evaporated in a vacuum oven at 300 mbar of absolute pressure and 40 °C overnight. In this way, nine different membranes were fabricated by modifying the content of IoNF from 0.2 to 8 wt%, and FIL from 19.2 to 40 wt% in the composite materials (see Table 3). All membranes were dense, non-porous flat films, whose thickness was measured with a Mitutoyo digital micrometer (MDC-25PX, accuracy ±1 μm). An average thickness of 80 ± 10 μm was obtained from nine measurements at different points of the membrane.

The IoNFs, CILPMs and MMMs prepared in this work were thoroughly characterized and the results described in Part I of this work. In particular, the properties of thermal stability, intermolecular interactions, glass transition temperature, morphology, roughness, and smoothness parameters, were experimentally determined to explore the effect of xGnP-IL fillers on the pristine Pebax^®^1657 sample, these parameters were determined by FTIR, TGA, DSC, STEM, and WLOP.

### 2.2. Gas Permeation: Mixed Gas Conditions

**Figure 1:**
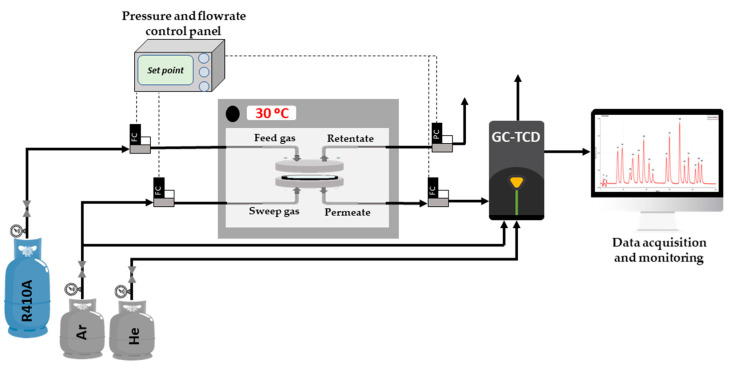
Schematic representation of the permeation setup.

Permeation experiments were performed in continuous operation by feeding the refrigerant mixture R410A inside a custom-made stainless steel permeation cell (see Figure 1). Pressure was controlled thanks to a back-pressure regulator placed in the retentate side of the permeation cell, while an argon stream (4 cm^3^_STP_·min^−1^ and 1 bar) was used as sweep gas in the permeate side. Concentration under steady state conditions, of R32 and R125, was measured with a gas chromatograph (Agilent 490 micro GC, Barcelona, Spain, equipped with a Pora Plot U column and a thermal conductivity detector). Temperature of the permeation tests was kept constant at 30 °C and the separation performance of the membranes was studied at 1.86 and 4.3 bar of feed pressure.

Gas permeability through the membrane was calculated according to Equation (1):

(1) $$P_{i}=\frac{Q_{i}\cdot \;\delta }{A\cdot \;\left({\hat{f}}_{R,i}-{\hat{f}}_{P,i}\right)}$$

where $P_{i}$ is the permeability of gas i through the membrane, $Q_{i}$ is the transmembrane flux of component i, calculated as the experimental concentration of the gas in the permeate stream multiplied by the permeate flowrate, δ and A are membrane thickness and area, respectively, ${\hat{f}}_{R,i}$ and ${\hat{f}}_{P,i}$ are the fugacity of component i in the gas mixtures of the retentate and permeate streams, respectively. Accordingly, ${\hat{f}}_{i}$ was calculated with Equation (2):

(2) $${\hat{f}}_{i}={\hat{\emptyset }}_{i}\cdot p_{i}$$

where ${\hat{\emptyset }}_{i}$ corresponds to the fugacity coefficient of component i in the R410A mixture, calculated with REFPROP property method of Aspen Plus, and $p_{i}$ is the partial pressure of gas i. In addition, the separation factor SF for each material tested was calculated according to Equation (3):

(3) SF= xR32pxR125pxR32fxR125f

where $x_{R32}$ and $x_{R125}$ are the mole fractions of R32 and R125 in the gas phase and the superscripts p and f stand for the permeate and feed side of the membrane, respectively.

### 2.3. Gas Sorption Measurements

Gas permeability (*P*) in polymer-based dense membranes can be described as the product of a solubility coefficient (*S*) and a diffusivity coefficient (*D*), according to the solution-diffusion model (Equation (4)). Therefore, the experimental determination of the solubility coefficient provides highly relevant information to understand the overall mechanism of gas permeation in the materials studied:

(4) P=S·D

**Figure 2:**
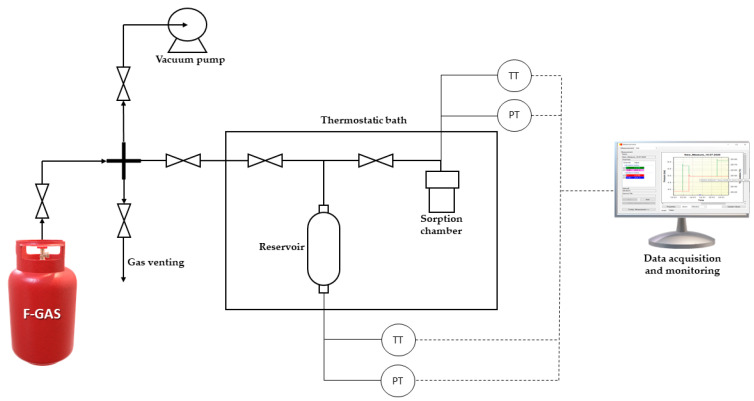
Schematic representation of the dual volume pressure decay experimental system used in this work.

The gas sorption in the prepared MMM materials was measured in an experimental setup based on the dual volume pressure decay method (see Figure 2). First, a sample of dense film membrane (~3 g) was rolled up and sandwiched between stainless steel mesh spacers, then placed inside the sorption chamber (22 mL stainless steel Parr reactor). Prior to each sorption test, the dense film was subjected to high vacuum and 30 °C for 24 h to desorb residual solvent and moisture. In each test, the F-gas (R32, or R125) was loaded into the reservoir (50 mL stainless-steel cylinder) at the desired pressure. Meanwhile, the sorption chamber remained at near-vacuum absolute pressure (<0.5 mbar). Once both pressure and temperature, measured and registered online over time with absolute pressure sensors (Keller PAA-33X series, 0.02 % accuracy at full scale), remained constant in the reservoir, the valve connecting both sections was opened and the sorption process occurred spontaneously until equilibrium was reached (no pressure changes for 30 min). To obtain a complete sorption isotherm, the process was repeated by loading the reservoir at a higher pressure in each stage.

Therefore, the number of sorbed moles of F-gas in each equilibrium step (Equation (5)) $n_{i,j}$ is calculated as the difference between the gas moles initially loaded in the reservoir and the remaining (not absorbed) after reaching equilibrium:

(5) $$n_{i,j}=\rho_{\left(i,j,R\right)}\cdot V_{R}+\rho_{\left(i,j-1,S\right)}\cdot \left(V_{S}-V_{F}-V_{M}\right)-\rho_{\left(i,\;j,S\right)}\cdot \left(V_{R}+V_{S}-V_{F}-V_{M}\right)$$

where $\rho_{\left(i,j,R\right)}$, $\rho_{\left(i,j-1,S\right)}$ and $\rho_{\left(i,\;j,S\right)}$ are the molar densities (mol·L^−1^) of gas i in the reservoir, in the sorption chamber at the previous equilibrium conditions, and in the total available volume after reaching the new equilibrium conditions, respectively. Molar density values have been determined at their corresponding pressure and temperature conditions with the REFPROP property method available in Aspen Plus. $V_{R}$, $V_{S}$, $V_{F}$, and $V_{M}$ are the volume of the reservoir, sorption chamber, dense film, and stainless-steel mesh spacers, respectively. Therefore, the total sorbed gas moles of component i in the polymer for each step $n_{i,total}$ are then calculated as the moles sorbed in the last step plus the accumulated moles sorbed in the previous k steps (Equation (6)):

(6) $$n_{i,total}=n_{i,j}+\overset{j-1}{\underset{k=1}{\sum }}n_{k}$$

Equation (7) shows the calculation of gas concentration ($C_{i,eq}$, cm^3^ (STP) gas·cm^−3^ polymer) at each equilibrium step:

(7) $$C_{i,eq}=\frac{22414\cdot n_{i,\;total}}{V_{F}}$$

## 3. Results and Discussion

### 3.1. R32 and R125 Permeability in Mixed Gas Conditions

**Figure 3:**
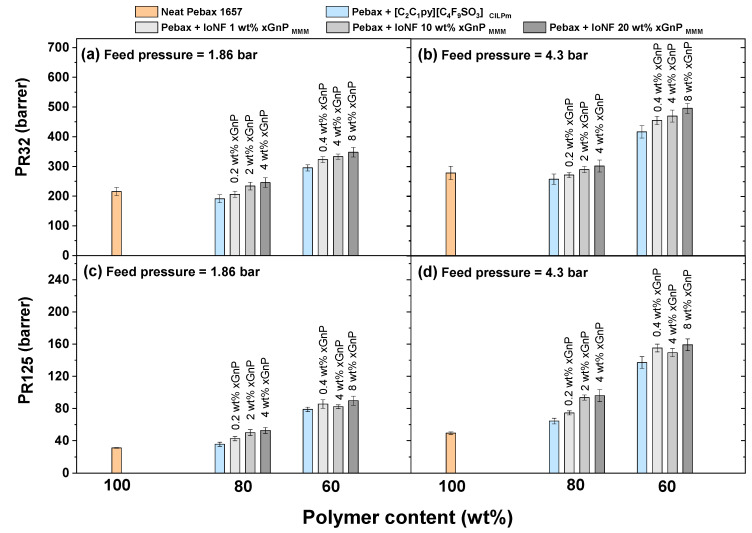
R32 (**a**,**b**) and R125 (**c**,**d**) mixed-gas permeability as a function of polymer content in the polymer, CILPMs and MMMs used in this work. Temperature = 30 °C, R410A feed pressures = 1.86 bar (**a**,**c**) and 4.3 bar (**b**,**d**).

The gas permeability of both R32 and R125, under mixed gas feed conditions and 30 °C, are shown in Figure 3 at different R410A feed pressures (1.86 and 4.3 bar) through all membranes prepared in this work (i.e., neat polymer, CILPMs and MMMs). As can be seen, the smallest molecule R32 (Chung diameter (R32) = 4.02 Å) permeates significantly faster than R125 (Chung diameter (R125) = 4.82 Å) in all the membranes tested. These results confirm the trend that has been already reported in a previous study regarding the performance of Pebax^®^1657-based CILPMs to separate R32 and R125 [23] and support the validity of this type of material for the selective recovery of R32.

With regard to the effect of the FIL [C_2_C_1_py][C_4_F_9_SO_3_] on the separation performance of the Pebax^®^ 1657-based CILPMs, it is first noted that in membranes with 20 wt% FIL, R32 permeability undergoes a slight decrease compared to the pure polymer upon both feed pressures tested (−11.3 % at 1.86 bar and −7.64 % at 4.3 bar). Particularly, at 4.3 bar, R32 permeability decreases from 279.3 barrer in the neat polymer to 257.9 barrer in the 20 wt% FIL composite membrane. In contrast, a moderate increase is noticed regarding R125 permeability (13.8% at 1.86 bar and 30.5% at 4.3 bar), which increases from 49.3 barrer in the neat polymer to 64.3 barrer in the 20 wt% FIL CILPM, working at 4.3 bar feed pressure. Furthermore, when FIL concentration in the CILPM increases to 40 wt%, the enhancement of gas permeability is much more notorious on both R32 (36.8% at 1.86 bar and 49.4% at 4.3 bar) and R125 (152% at 1.86 bar and 178% at 4.3 bar).

On the one hand, the increase of F-gas permeability with feed pressure in this kind of rubbery dense films confirms the highly sorbing nature of F-gases, which exhibit a remarkable plasticizing effect on the rubbery Pebax^®^ polymer, analogous to that observed with CO_2_ [29,54,55,56]. Furthermore, when ILs are integrated into Pebax^®^1657-based CILPMs, the increase on gas permeability at higher IL loadings can be attributed to the enhancement of both gas diffusivity and solubility. Indeed, since the permeability of both R32 and R125 dramatically increase at higher R410A feed pressures, these results suggest that the FIL [C_2_C_1_py][C_4_F_9_SO_3_] causes an extraordinary enhancement on F-gas solubility with respect to the neat polymer Pebax^®^1657, which in turn is in line with the observed behavior for the absorption of fluorinated gases on this FIL [12].

Concerning the MMMs fabricated in this work, the presence of the xGnP in the ternary membrane systems (polymer, FIL and xGnP) leads to an enhancement of R32 and R125 permeabilities with respect to the CILPMs made with an analogous FIL content. First, at 1.86 bar feed pressure, for the 80 wt% Pebax-based CILPMs and MMMs, R32 permeability gradually increases with xGnP content from 191.2 barrer in the 80 wt% CILPM to 245.5 barrer in the 4xGnP-FIL-80Pebax MMM, which represents an increment of 28.4%. In the case of R125, a 48% permeability increase is noticed between the 4xGnP-20IoNF-80Pebax membrane (52.7 barrer) and its comparable 20FIL-80Pebax CILPM (35.6 barrer). Moreover, the increase of feed pressure to 4.3 bar results in notable permeability enhancements., e.g., the R32 permeability varies from 257.9 barrer in the 80 wt% Pebax CILPM to 302.2 barrer in the 4xGnP-20IoNF-80Pebax MMM. In addition, when the amount of polymer in the dense film is reduced to 60 wt% of Pebax^®^1657 and the additive consists of 40 wt% of either FIL or IoNF, both R32 and R125 permeability values exceed those corresponding to the membranes with 80 wt% polymer. Particularly, the maximum permeability values obtained in this work for R32 and R125, 496.1 barrer and 159.1 barrer respectively, correspond to the 8xGnP-40IoNF-60Pebax MMMs upon 4.3 bar of feed pressure, meaning a 17.8% and 13.6% permeability increment, with respect to the analogous 40FIL-60Pebax CILPM.

Taking into consideration the abovementioned effects, it is worth mentioning that the most significant modifications on F-gas permeability with respect to the neat polymer are caused to a greater degree by the presence of IL rather than by the presence of xGnP, regardless of the concentration of inorganic filler. These trends are in accordance with the literature, for instance, an enhanced CO_2_ permeation was reported on 1-(3-aminopropyl)-3-methylimidazolium bromide and GO Pebax^®^ based MMMs [57], and similarly for pervaporation applications, an improved absorption of butanol by the presence of the IL n-octylpyridinium bis(trifluoromethyl)sulfonylimide in GO-IL Pebax-based MMMs has been reported [58]. On the other hand, the slight increase of F-gas permeability noticed at higher xGnP concentrations may be due to the fact that xGnP is a sort of material that does not exhibit strong interactions with the polymer chains and tends to agglomerate as stacked laminates. Accordingly, the transport of gas molecules may occur through interstitial hollow regions located between the polymer chains and the inorganic filler or through regions of lower packing density caused by the expansion of free volume by the inorganic filler [38,59,60].

### 3.2. R32/R125 Separation Factor

In order to develop attractive materials to optimize the separation of this type of refrigerant gases, it is envisaged that FILs can be good candidates given their high solubility to F-gases. To this purpose, it is crucial to further investigate their effect on the separation performance in addition to the previously observed increase in permeability.

**Figure 4:**
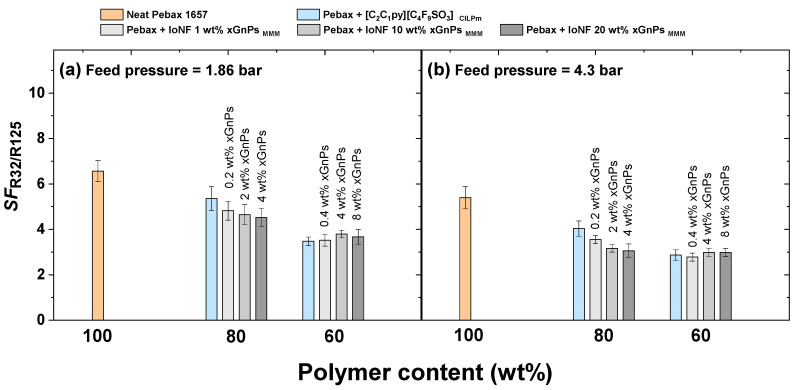
R32/R125 separation factor in neat Pebax^®^1657, CILPMs and MMMs at 30 °C and R410A feed pressures of 1.86 bar (**a**) and 4.3 bar (**b**).

In this regard, Figure 4 shows the separation factor (Equation (3)) as a function of the mass percentage of polymer, at the two pressures of R410A tested (1.86 and 4.3 bar) and for the nine membranes studied in this work. The results show that the presence of the FIL reduces the selectivity performance of CILPMs with respect to the pure polymer. This trend is also confirmed upon higher concentrations of IL in the CILPM and at higher pressures. This is in very good agreement with the fact that the functional properties of CILPMs prepared with highly fluorinated ILs result in very noticeable increases in gas permeability, yet at the expense of reducing the separation factor. For instance, a reduction of the selectivity of a Pebax^®^1657-based CILPM prepared with [C_2_C_1_im][Tf_2_N] with respect to neat Pebax^®^1657 has been previously observed for the separation of a R410A mixture [23]. For CILPMs-based separations of other gas mixtures, such as CO_2_/CH_4_ or CO_2_/N_2_, the IL [C_4_C_1_im][Tf_2_N], which exhibits a high degree of fluorination, also presents the highest values of CO_2_ permeability at the expense of a selectivity decline [61]. Concerning the effect of the working pressure, the decrease in the separation factor as the pressure increases is a quite frequent phenomenon, especially in high sorbing rubbery polymers with high plasticizing behavior under mixed gas experimental conditions [22,23,62].

Two trends are observed in relation to the effect of xGnP concentration within the MMMs. First, for membranes consisting of 80 wt% polymer and 20 wt% IoNF, a gradual decrease in the separation factor is observed as the content of xGnP increases. Accordingly, the separation performance follows the order 20FIL-80Pebax ($SF_{1.86bar}$ = 5.4; $SF_{4.3bar}$ = 4.0) > 0.2xGnP-20IoNF-80Pebax ($SF_{1.86bar}$ = 4.8; $SF_{4.3bar}$ = 3.6) > 2xGnP-20IoNF-80Pebax ($SF_{1.86bar}$ = 4.6; $SF_{4.3bar}$ = 3.2) > 4xGnP-20IoNF-80Pebax ($SF_{1.86bar}$ = 4.5; $SF_{4.3bar}$ = 3.1). This loss of selectivity, added to the increase in permeability, occurs in MMMs in which the agglomeration of the inorganic fillers causes the formation of interfacial defects in the form of nonselective voids that lead to lower separation factors [63,64]. Secondly, when the amount of IoNF increases in the membranes to 40 wt%, the separation factor does not present significant modifications in the whole range of xGnP compositions studied. According to Figure 4, at 1.86 bar, $SF^{40FIL-60Pebax}$ = 3.5; $SF^{0.4GNs-40IoNF-60Pebax}$ = 3.5; $SF^{4GNs-40IoNF-60Pebax}$ = 3.8; $SF^{8GNs-40IoNF-60Pebax}$ = 3.7, whereas at 4.3 bar, $SF^{40FIL-60Pebax}$ = 2.9; $SF^{0.4GNs-40IoNF-60Pebax}$ = 2.8; $SF^{4GNs-40IoNF-60Pebax}$ = 3.0; $SF^{8GNs-40IoNF-60Pebax}=3.0$. In this case, these results suggest that the higher IL concentration in the hybrid membranes could play a crucial role in minimizing the generation of defects between the NGs and the polymer chains. According to the literature, during permeation of this type of gases in MMMs, if interfacial nonselective voids are filled with IL, gas transport is likely to occur through extra FFV or lower packing zones generated by the xGnP. Therefore, gas permeability can be increased without resulting in a significant reduction of the separation factor [65].

**Figure 5:**
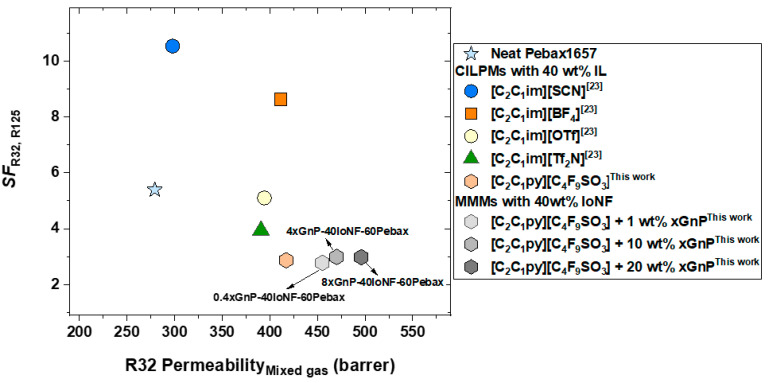
Summary of $SF_{R32,R125}$ vs. R32 mixed gas permeability (30 °C and 4.3 bar R410A feed pressure) for different CILPMs and MMMs with 60 wt% Pebax^®^1657.

An overview of the separation performance of the MMMs prepared in this work is presented in Figure 5 in the form of a Robeson type diagram ($SF_{R32,R125}$ vs. R32 permeability under mixed gas conditions). Here, the separation factor of the MMMs manufactured with 60 wt% Pebax^®^1657 and 40 wt% of IoNF is compared, at the same R410A feed pressure of 4.3 bar and polymer content, with that of the CILPMs previously studied. It is first observed that the IL used in the present work notably improves R32 permeability with respect to the ILs [C_2_C_1_im][SCN], [C_2_C_1_im][BF_4_], [C_2_C_1_im][OTf], and [C_2_C_1_im][Tf_2_N] at the expense of a lower selectivity. Furthermore, considering that the addition of xGnP to the MMMs improves gas permeability while keeping $SF_{R32,R125}$ stable as previously stated, we hypothesize that MMMs prepared with IoNF based on more selective ILs such as [C_2_C_1_im][SCN] and [C_2_C_1_im][BF_4_] would allow pushing the limits of these hybrid membranes toward more attractive R32 permeability values, similar to those observed in this work, while providing high separation factors. This would require further dedicated research to study the surface behavior of these ILs and their ability to exfoliate the xGnP and produce stable IoNFs as efficiently as the FIL [C_2_C_1_py][C_4_F_9_SO_3_].

**Figure 6:**
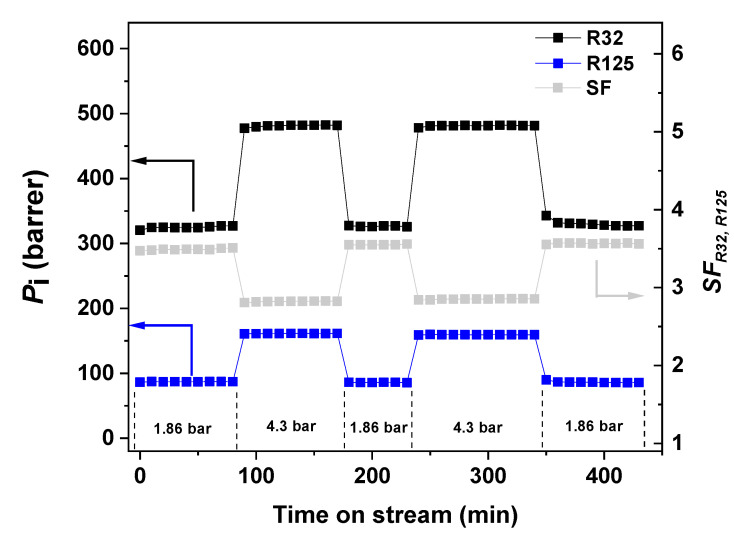
R32 and R125 permeability and $SF_{R32,R125}$ vs. time on stream at 30 °C through MMM 4xGnP-40IoNF-60Pebax. Feed gas: R410A mixture at 1.86 bar and 4.3 bar.

Another important aspect to study in this type of hybrid materials is their separation performance over a sustained period of time under different operating pressures. Figure 6 shows the permeability of R32 and R125 as well as $SF_{R32,R125}$ versus time on stream for 430 min through the MMM 4xGnP-40IoNF-60Pebax. It is observed that under these cyclic changes of pressure, the MMM exhibits a robust behavior without changes in the gas permeability and separation factor observed at each pressure.

### 3.3. R32 and R125 Solubility Behavior

To elucidate the contribution of the FIL and the xGnP on the F-gas sorption ability of the CILPMs and MMMs studied in this work, the solubility behavior toward R32 and R125 has been evaluated individually in three different membranes in the pressure range from 1 to 6 bar. The membranes chosen for comparison were neat Pebax^®^1657, 40FIL-60Pebax CILPM with and 4xGnP-40IoNF-Pebax60 MMM.

**Figure 7:**
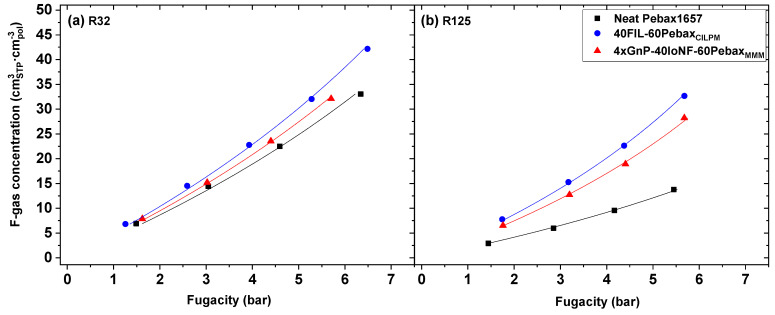
Membrane gas sorption equilibrium data. The concentration of R32 (**a**) and R125 (**b**) adsorbed in the membrane phase as a function of solute fugacity in the gas phase. Solid lines correspond to the fit of experimental data to the Flory–Huggins model (Equation (8)).

Figure 7a,b illustrate the sorption isotherms of R32 and R125, respectively, as a function of gas fugacity. The Flory–Huggins (FH) model (Equation (8)) was used to fit the experimental data. This model is able to accurately describe sorption isotherms of highly-sorbing species, such as vapors or condensable gases, that frequently form solute clusters during the sorption process into rubbery polymers [66]. This type of behavior is characterized by sorption isotherms that are convex to the fugacity axis as those shown in Figure 7. An additional advantage of using the FH model is that it provides insight on the polymer-gas interactions through the interaction parameter χ [67]. In particular, values greater than 2 are an indication of small polymer-gas interactions, and values lower than 0.5 are indicative of very strong interactions [68].

(8) $$\ln\frac{f}{f_{sat}}=\ln\emptyset +\left(1-\emptyset \right)+\chi {\left(1-\emptyset \right)}^{2}$$

In Equation (8), f and $f_{sat}$ are the gas fugacity and saturation fugacity at the corresponding equilibrium pressure and temperature, and ∅ is the volume fraction of penetrant dissolved in the polymer. In addition, Equation (9) shows the calculation of parameter ∅ from the equilibrium gas concentration (Equation (7)) and the F-gas liquid molar volume $\bar{V}$ (cm^3^·mol^–1^), obtained from REFPROP, at the sorption equilibrium temperature [67]:

(9) $$\emptyset =\frac{C_{i,eq}\bar{V}}{22414+C_{i,eq}\bar{V}}$$

Therefore, the solid lines in Figure 7, which represent the estimated values of the FH model corresponding to each membrane studied, show a good coefficient of determination (r^2^ > 0.995) for the description of R32 and R125 sorption behavior over pressures ranging from 1 to 6 bar. Besides, the different FH interaction parameters χ obtained are shown in Table 4.

The results obtained reveal and confirm that the presence of the FIL [C_2_C_1_py][C_4_F_9_SO_3_] notably increases both R32 and R125 solubility in the CILPM compared to the neat Pebax^®^1657 membrane. This is further corroborated by the values of χ parameter of the FH model, which significantly decreases upon addition of FIL ($\chi_{NeatPebax}^{R32}\;$= 0.902; $\chi_{40FIL-60Pebax}^{R32}$ = 0.727; $\chi_{NeatPebax}^{R125}$ = 1.22; $\chi_{40FIL-60Pebax}^{R125}$ = 0.512), thus indicating that stronger interactions are taking place between the penetrants and the composite membrane after addition of the FIL. However, the solubility difference between R32 and R125 become narrower in the CILPM than in the pure polymer, confirming the role played by the FIL in the composite membrane, i.e., increasing the permeability of both gases at the expense of decreasing the solubility selectivity. In the case of xGnP addition, both R32 and R125 sorption isotherms related to the 4xGnP-40IoNF-60Pebax MMM are located between the isotherms corresponding to the neat polymer and the CILPM. This fact may be attributed to a weak affinity between the F-gases and the xGnP, as reflected by the slight increase observed in χ parameter, which in turn occupy spaces within the polymeric matrix and block the solubility of both R32 and R125. Therefore, considering that gas permeability slightly increases through MMMs despite the solubility loss caused by the addition of xGnP, this effect must be due to improvements in R32 and R125 gas diffusivity through the hybrid materials [69].

## 4. Conclusions

In the present work, we explore for the first time the separation potential of Pebax-based MMMs prepared with ioNanofluids (IoNF) for the selective recovery of the refrigerant gas R32 from the mixture R410A. The MMMs were manufactured by the solvent casting method using Pebax^®^1657 as the polymer matrix and stable IoNFs that combined the FIL [C_2_C_1_py][C_4_F_9_SO_3_] and xGnP in different proportions. From the study of the permeation properties, under mixed gas conditions and several R410A feed pressures, and the single gas solubility over a wide pressure range, it was possible to ascertain the role of the IL and the xGnP in enhancing the separation performance of the new hybrid membranes compared to that of the base polymer. In this sense, xGnP present little interaction with the studied gases (R32 and R125), acting as solubility blockers and exerting a modification of the internal structure of the membranes that mainly modifies the gas diffusivity. Overall, it was observed that at high FIL concentrations the addition of xGnP lead to a significant increase in the permeability of both gases without producing negative alterations in the R32/R125 separation factor. However, despite the high permeability results obtained, the resulting separation performance was affected by the relatively low selectively of the FIL selected to prepare the IoNF. In light of these results, it is expected that deepening in the dense film fabrication technique in order to minimize the random and stacked orientation of xGnP as well as studying novel IoNF prepared with more selective ILs could allow reaching very important advances in this specific field of refrigerant gas separations.

## Figures and Tables

**Table 1:** General properties of Pebax^®^1657MH grade [22].

Grade	Pebax^®^1657MH
Molecular structure of repeated unit	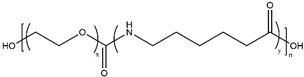
Density (g·cm^−3^)	1.14
Copolymer composition	60 wt% PEO, 40 wt% PA6

**Table 2:** Characteristics of [C_2_C_1_py][C_4_F_9_SO_3_] used in this work (density, viscosity, and molar volume values are given at 30 °C).

FIL Designation	[C_2_C_1_py][C_4_F_9_SO_3_]
Molecular structure	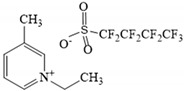
Molar mass (g·mol^−1^)	421.28
Density (g·cm^−3^) [53]	1.51
Viscosity (mPa·s) [53]	150.3
Molar volume (cm^3^·mol^−1^)	279.01
CAS No.	1015420-87-7

**Table 3:** Summary of the membranes fabricated in this work.

Type of Membrane	Membrane	Polymer Content (wt%)	FIL Content (wt%)	xGnP Content (wt%)
Polymeric	Neat Pebax	100	-	-
CILPM	20FIL-80Pebax	80	20	-
	40FIL-60Pebax	60	40	-
MMM	0.2xGnP-20IoNF-80Pebax	80	19.8	0.2
	2xGnP-20IoNF-80Pebax		18	2
	4xGnP-20IoNF-80Pebax		16	4
	0.4xGnP-40IoNF-60Pebax	60	39.6	0.4
	4xGnP-40IoNF-60Pebax		36	4
	8xGnP-40IoNF-60Pebax		32	8

**Table 4:** Fitted Flory–Huggins interaction parameter (χ ) of R32, and R125 in neat Pebax^®^1657, 40FIL-60Pebax CILPM and 4xGnP-40IoNF-60Pebax MMM. Experimental standard deviation < 5%.

Penetrant	Neat Pebax^®^1657	CILPM 40FIL-60Pebax	MMM 4xGnP-40IoNF-60Pebax
R32	0.902	0.727	0.813
R125	1.221	0.512	0.665

## Data Availability

The data presented in this study are available on request from the corresponding author.
